# Functional Lung Imaging during HFV in Preterm Rabbits

**DOI:** 10.1371/journal.pone.0048122

**Published:** 2012-10-30

**Authors:** Jordan Thurgood, Stuart Hooper, Melissa Siew, Megan Wallace, Stephen Dubsky, Marcus Kitchen, R. Aidan Jamison, Richard Carnibella, Andreas Fouras

**Affiliations:** 1 Department of Mechanical and Aerospace Engineering, Monash University, Melbourne, Victoria, Australia; 2 The Ritchie Centre, Monash Institute of Medical Research, Melbourne, Victoria, Australia; 3 School of Physics, Monash University, Melbourne, Victoria, Australia; University of Giessen Lung Center, Germany

## Abstract

Although high frequency ventilation (HFV) is an effective mode of ventilation, there is limited information available in regard to lung dynamics during HFV. To improve the knowledge of lung function during HFV we have developed a novel lung imaging and analysis technique. The technique can determine complex lung motion information *in vivo* with a temporal resolution capable of observing HFV dynamics. Using high-speed synchrotron based phase contrast X-ray imaging and cross-correlation analysis, this method is capable of recording data in more than 60 independent regions across a preterm rabbit lung in excess of 300 frames per second (fps). This technique is utilised to determine regional intra-breath lung mechanics of preterm rabbit pups during HFV. Whilst ventilated at fixed pressures, each animal was ventilated at frequencies of 1, 3, 5 and 10 Hz. A 50% decrease in delivered tidal volume was measured at 10 Hz compared to 1 Hz, yet at the higher frequency a 500% increase in minute activity was measured. Additionally, HFV induced greater homogeneity of lung expansion activity suggesting this ventilation strategy potentially minimizes tissue damage and improves gas mixing. The development of this technique permits greater insight and further research into lung mechanics and may have implications for the improvement of ventilation strategies used to support severe pulmonary trauma and disease.

## Introduction

Conventional ventilation (CV) is commonly used to support breathing in both newborn and adult patients. If applied incorrectly, CV can cause ventilator induced lung injury (VILI), due to atelectasis (repeated opening and closing of alveoli) or overdistention of lung tissue [1], [2], [3]. Ventilation with smaller tidal volumes (V_t_) has been shown to minimize lung damage [2], [3], [4], [5], [6]. As such, high frequency ventilation (HFV) may reduce VILI through the delivery of smaller volumes at higher ventilation rates, allowing increased minute volumes (product of tidal volume and frequency) and CO_2_ clearance [7], [8], [9]. Human [2], [5], [10], [11], [12] and animal [13], [14] studies indicate that HFV is an effective and safe mode of ventilation, however, there has been much inconsistency as to the specific HFV parameters that should be applied [15].

Typically during HFV inflations are delivered at 3 Hz to 15 Hz [16] using small tidal volumes that can potentially be less than the anatomical dead space [7], [17], [18]. Thus, the principal mechanism of gas exchange cannot be bulk gas transport, as occurs during normal respiration [19]. The underlying gas exchange mechanisms have been the subject of much debate [20], [21] and are not yet fully understood [19], [20], [22], [23]. It is proposed that increased minute volumes, along with enhanced gas mixing mechanisms, effectively and safely promote gas exchange during HFV [19], [20], [21], [24].

Although much research has focussed on optimizing HFV [25], [26], [27], [28], [29], [30], major improvements have been limited by a lack of knowledge of regional lung function during HFV. In particular, a regional understanding of tissue mechanics and gas transport is required to understand how the smaller respiratory units interact to effect efficient gas transfer [20], [21], [24]. Furthermore, the information must be obtained with sufficient temporal resolution to observe the dynamics within the respiratory cycle [31].

At the frequencies employed in HFV, imaging the lungs with sufficient temporal and spatial resolution is not possible with standard imaging approaches. Several techniques such as electrical impedance tomography (EIT), respiratory inductance plethysmography (RIP), magnetic resonance imaging (MRI) and X-ray computed tomography (CT) have been applied to investigate the lung during HFV. Whilst providing important information, each of these techniques possess specific limitations that restrict their ability to investigate lung dynamics during HFV. For instance, EIT [32] provides poor spatial resolution in addition to typically having temporal resolutions below 44 Hz [33], [34], [35], [36]. Although RIP can measure lung volume changes, it provides no spatial information on gas distribution within the lung [37], [38]. MRI and CT both offer higher spatial resolution than EIT [22], [39], [40], [41], [42], [43], but acquisition times at these higher spatial resolutions often require measurements to be made over multiple breath cycles [44], [45], [46] especially during HFOV conditions. Image blurring, due to a combination of lung motion and exposure times, has greatly limited the use of imaging to assess regional lung function with high spatial resolution [44].

Conventional (absorption based) X-ray imaging provides very poor levels of contrast in the lung. However, the lung (with its many tissue/air boundaries) is ideal for a method known as phase contrast X-ray imaging and for the lung this method provides images of high contrast and high detail [47]. Synchrotron X-ray sources provide highly coherent monochromatic X-rays that are well suited to phase contrast imaging. By combining this imaging method with velocimetry techniques, X-ray velocimetry was developed [48], [49]. X-ray velocimetry can non-invasively and accurately measure complex patterns of motion in opaque samples [48], [50], [51]. The application of X-ray velocimetry to the lungs results in vector fields defining the speed and direction of local lung tissue motion between consecutive frames, providing information on local lung mechanics with high spatial and temporal resolution [49], [52], [53]. Our aim was to regionally analyse the effect of ventilation frequency on lung tissue behaviour during HFV for the first time.

## Materials and Methods

### Ethics Statement

All animal procedures were approved by the SPring-8 Animal Care Committee and Monash University’s School of Biomedical Science’s Animal Ethics Committee. All studies were conducted in experimental hutch 3 of beamline 20B2, in the Biomedical Imaging Centre at the SPring-8 synchrotron in Japan.

### Animal Preparations

Pregnant New Zealand white rabbits (preterm age of 28 days of gestation; term = 32d) were anaesthetised (propofol; i.v.; 12 mg/kg bolus), intubated and anaesthesia was maintained (isoflourane inhalation 1.5–4%). Pups were delivered by caesarean section, sedated (Nembutal; 0.1 mg, i.p.) and intubated with an endotracheal (ET) tube (18G) before the umbilical cord was cut. The pup was positioned upright in a warm water-filled plethysmograph (head out) located in the X-ray beam within the imaging hutch as previously described [54]. The ET tube was connected to the ventilator and animals were ventilated using a custom, pressure controlled small-animal ventilator [55]. Animals were ventilated using CV to fully aerate the lung and establish a functional residual capacity before HFV commenced. Airway pressure was oscillated between a positive end expiratory pressure (PEEP) of 9 cmH_2_O and a peak inspiratory pressure (PIP) of 21 cmH_2_O with ventilation frequencies of 1, 3, 5 and 10 Hz. Airway pressure and air flow were measured at the mouth opening using a pressure sensor and a pneumotach, respectively. Tidal volumes were calculated by integrating the flow signal.

### Imaging Setup and Parameters

**Figure 1:**
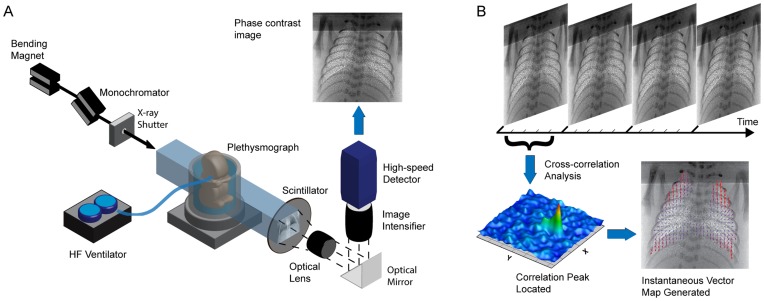
Method and apparatus for acquisition of high spatiotemporal resolution *in vivo* information. A) Experimental setup designed for the acquisition of synchrotron based phase contrast X-ray lung images with high spatial and temporal resolution (image modified from [52]). The set-up allows the acquisition of *in vivo* images with enhanced lung speckle, which provides detail necessary for a cross-correlation analysis. B) Cross-correlation analysis of images allows the determination of the displacement of lung tissue within each image interrogation region. The statistically most likely displacement for each respective window is determined as the displacement of lung tissue within that interrogation region. This is performed across the entire lung image, thus generating a vector field incorporating the entire lung.

The highly coherent and bright X-ray source at beamline 20B2 offers the potential to perform *in vivo* studies with high spatial and temporal resolution. Radiation generated at the bending magnet was filtered to provide monochromatic X-rays (24 keV) and was directed through the pup in the water-filled plethysmograph (Figure 1). Images were acquired using a scintillator (Hamamatsu P43), image intensifier (Lambert II18 Gen3) and high-speed camera (IDT Y4; 1016×1016 pixel sensor). The propagation distance from the animal to the detector was 3 m. The combined effect of sensor pixel size and optical magnification yields an effective pixel size of 20.6 µm.

### Image Analysis

All images were flat field corrected to improve image quality [56]. A procedure to remove low frequency artefacts, such as intensity gradients due to the use of a cylindrical water-filled plethysmograph, was also applied. In this procedure all rows in the image were scaled to have the same average pixel intensity, which was then successively applied to scaling of the columns, and repeated.

**Figure 2:**
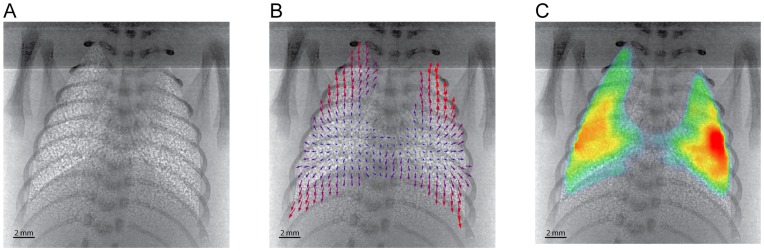
Information determined from *in vivo* lung imaging and analysis technique. A) Average image of 10 phase contrast images of a rabbit pup taken with 2 ms exposures at a rate of 300 fps (1016×1016 pixels). The image shows the enhanced lung structure attained with this imaging technique. B) Vector map showing instantaneous lung motion between 2 subsequent images. A cross-correlation window size of 64×64 px and 75% window overlap was used, with only a quarter of the >1000 vectors being shown for clarity. C) Map coloured according to the local lung tissue expansion at an instant in time, determined between 2 subsequent frames; red being largest and blue being smallest expansion values respectively.

The high contrast lung speckle, as seen in Figure 2A, Video S1, Video S2, provides a rich level of detail for cross-correlation analysis of motion at high spatial resolution [53]. Bone structure within the images was minimised via a band-pass spatial frequency filter to improve image quality and minimise errors in the cross-correlation analysis of the lung motion. A mask image was created which constrained the X-ray velocimetry measurements to regions of lung tissue.

### X-ray Velocimetry Analysis

A cross-correlation analysis is performed on the images by first dividing the image into regions, known as interrogation windows. An FFT-based cross-correlation is then performed on each interrogation window between subsequent frames. The peak of the resulting cross-correlation is the modal value for displacement within that particular window at that point in time. Window sizes are chosen to improve resolution whilst minimising errors. Large windows include more lung speckle thus providing improved signal to noise ratio (SNR). Smaller windows provide better resolution at the cost of the SNR. Furthermore, too small a window and the speckle may move outside of the window, thus creating additional errors. Noisier images require larger windows to get a statistically significant answer, therefore the spatial resolution decreases with an increase in image noise via the larger windows required for analysis. For a more in depth understanding of the process refer to [57].

To determine any motion of the animal as a whole a cross-correlation analysis was performed using large interrogation windows that included the bone structure of the animal; this was subtracted from the motion of the lungs. The X-ray velocimetry measurements were performed using in-house software as used in [52] and as described in detail in [57] with a detailed error analysis in [58]. The cross-correlation utilised an interrogation window size of 64×64 pixels and 75% window overlap. An iterative approach was applied with 128×128 pixel windows used first to get an approximation of the local motion; this information was then used in the subsequent 64×64 analysis to increase dynamic range and spatial resolution (see for example [48]). The analysis provided a vector spacing of 330 µm resulting in over 1000 measurements in total across the lung at each time point. The result can be seen in Figure 2B as a vector map.

Instantaneous local lung tissue expansion has been shown to be derived from the spatial derivatives of the velocity field [52]. Figure 2C shows a map of the local lung tissue expansion between two successive frames. Expansion activity is defined as the root-mean-square (RMS) of the local tissue expansion through time. Expansion activity includes the degree of lung expansion activity during both inspiration and expiration phases. The 2D X-ray velocimetry measurements at each location are a representation of the motion of lung tissue through the entire depth of the lung at that particular location.

Local tissue expansion was integrated across the lung to give instantaneous respiratory flow. Respiratory flow was integrated through time to give respiratory volume. Respiratory volume was calibrated using the method described in [52] to determine the tidal volume in millilitres.

## Results and Discussion

**Figure 3:**
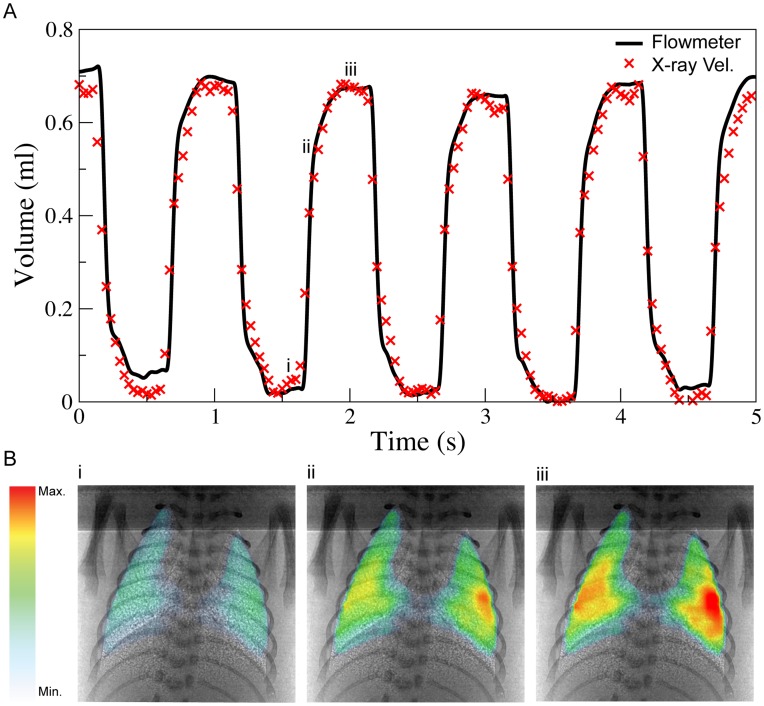
Temporal measurement of lung activity via traditional methods and the X-ray velocimetry technique. A) Time plots showing the tidal volume delivered as measured with a flowmeter and X-ray velocimetry integrated expansion. The X-ray velocimetry integrated expansion was calibrated to volume according to the method of Fouras et. al [52]. The coefficient of determination between the flowmeter and the X-ray velocimetry volume was >0.96. The animal was being ventilated at PEEP of 9 cmH2O, PIP of 21 cmH2O and a frequency of 1 Hz. B) X-ray velocimetry integrated expansion maps calculated from the end expiration to early inspiration (i), mid-inspiration (ii) and end-inspiration (iii). The X-ray velocimetry integrated expansion maps correlate to the time indicated by the (x) symbols on the X-ray velocimetry time plot in A). It can clearly be seen that as inspiration progresses a greater amount of lung tissue expansion has occurred and the expansion maps are able to show where the changes have occurred with high spatial resolution.

Figure 3A shows respiratory volume measured using our imaging technique for a ventilation rate of 1 Hz. This measurement was found to faithfully reproduce the tidal volume measured via a pnuemotach (coefficient of determination >0.96). Figure 3B presents maps of accumulative regional lung expansion calculated with respect to the onset of inspiration at three time points that are labelled in Figure 3A. Each data point on the respiratory volume trace in Figure 3A is the sum of more than 1000 locations across the lung, as seen in the expansion maps in Figure 3B. This data clearly demonstrates the spatial and temporal resolution that can be achieved using this technique and the potential for using it to assess how different ventilation strategies alter the distribution of ventilation within the lung.

**Figure 4:**
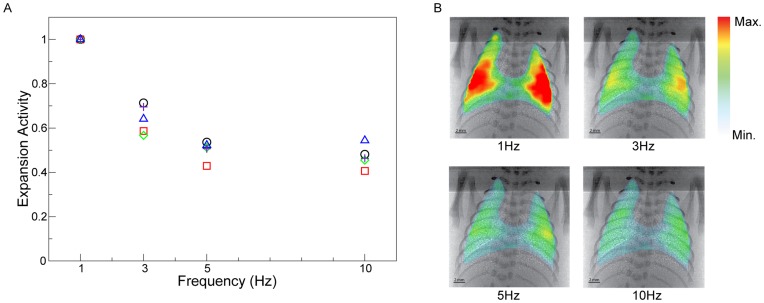
Effect of frequency on expansion activity. A) Effect of frequency on expansion activity normalised to the activity at 1 Hz for each animal to account for inter-animal effects. Each animal is marked by a different symbol. Overall we see a decrease in the magnitude of expansion activity for each respiration cycle as the ventilation frequency is increased. B) Expansion maps showing the effect of ventilation rate on activity of the lung throughout the ventilation cycle. As the ventilation rate is increased it can be seen that the expansion activity decreases. Expansion maps shown have over 1000 measurement locations in each map. Expansion activity, as shown here, is a dimensionless quantity.

Increasing inflation frequency whilst maintaining fixed end-inspiration and end-expiration pressures reduced the magnitude of expansion activity (Figure 4A). Expansion activity was normalised to that delivered at 1 Hz to account for inter-animal variability. There was an average decrease in expansion activity of 50% at 10 Hz compared to 1 Hz. The frequency dependent decrease in expansion activity is due to the decrease in inspiration time which limits the duration over which gas can flow into the lung, as previously described [59]. A decrease in expansion activity is representative of a decrease in tidal volume which is thought to be a necessary and an advantageous outcome of higher ventilation frequencies [2].

**Figure 5:**
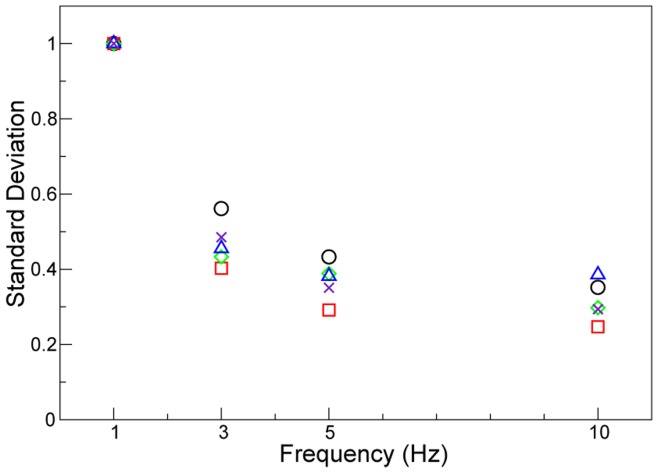
Spatial standard deviation of expansion plotted against ventilation frequency. Spatial standard deviation of expansion activity plotted against ventilator frequency, normalised by the value at 1 Hz for each animal to account for inter-animal effects. There is a decrease in expansion activity variation as the rate of ventilation is increased. Values for the 5 animals are shown and are normalised to the 1 Hz value to minimize inter-animal effects. Each animal is marked by a different symbol. A higher degree of spatial variation of expansion at lower frequencies suggests that with a longer time available for gas flow the distribution of expansion is primarily dependant on the local lung compliance. At the higher frequencies, with a limited time for expansion to occur, the expansion activity is more consistent across the lung as shown by a smaller standard deviation in the activity.

An important finding of this study is that with increasing frequency the magnitude of lung tissue expansion activity throughout the lung markedly decreased (Figure 4B), simultaneously with increasing uniformity of ventilation throughout the lung (Figure 5). These findings demonstrate that at higher frequencies the lung behaves more uniformly which likely protects areas of lung tissue from over-distension. When ventilated at lower frequencies, lung tissue has time to expand according to its local compliance. However at rates of ventilation that are faster than the time constant of the lung tissue, the effect of localised differences in tissue compliance is minimised resulting in more uniform inflation.

**Figure 6:**
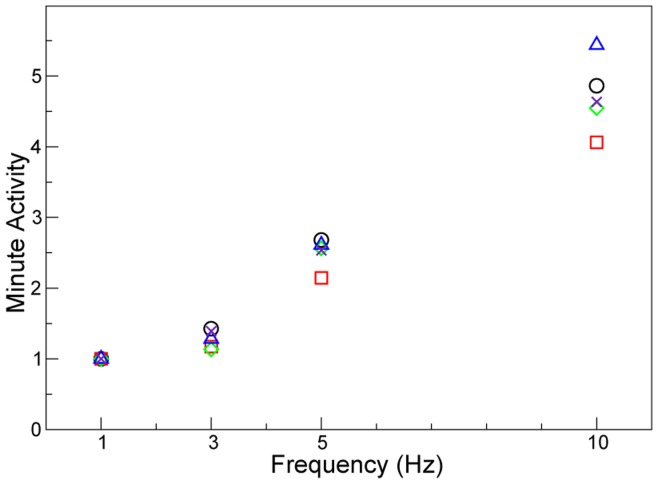
Effect of frequency on the minute activity. The normalised minute expansion, calculated as X-ray velocimetry expansion activity multiplied by the frequency (in breaths per minute), is shown for the 5 animals and is normalised around the 1 Hz value to account for inter-animal effects. Each animal is marked by a different symbol. This term, analogous to the minute volume, shows the extent of expansion activity that occurs at each ventilation rate over the same interval of time. As the rate of ventilation is increased it can readily be seen that the total amount of expansion activity also increases, thus providing potential for improved airflow and gas mixing.

The minute activity, calculated as the expansion activity multiplied by the number of cycles per minute, was found to increase as the rate of ventilation increased (Figure 6) despite decreased tidal volumes. At 10 Hz, there was approximately a 5-fold increase in minute activity compared to 1 Hz. Therefore, over the same duration, more gas would be exchanged with the animal at the higher frequencies than would at the lower frequencies, despite the use of smaller tidal volumes. This high amount of gas transport activity allows for several of the postulated HFV gas exchange mechanisms [20], [21], [24] to exist. The tidal volumes delivered varied from ∼0.8 ml at 1 Hz to ∼0.4 ml at 10 Hz. Siew et al. [60] have shown that for animals of this breed and weight the respiratory dead space is approximately 0.08 ml. Therefore the total effect respiratory dead space would not change the trends observed. Despite the use of shorter ventilation cycles, HFV was capable of providing fresh gas to the animals with each cycle, whilst minimizing lung tissue excursion per cycle. Further evidence of this can be seen in Video S1 and Video S2.

Current limiting factors to be overcome are mostly based around the translation of this imaging technology towards a laboratory or clinical environment. However, with a steady advance in X-ray source technology for example the advent of the liquid metal jet anode source [61], translation of this imaging method out of the synchrotron environment appears to be plausible.

### Conclusion

Previously, global measures were the only methods with suitable temporal resolution for investigating HFV. The use of this 2D X-ray velocimetry technique allows for a regional and intra-breath analysis of HFV *in vivo*. We hypothesize that during HFV the increase in minute lung activity with a decrease in tidal volume would result in an increase in gas mixing and gas exchange whilst potentially minimizing lung damage by preserving uniformity of lung tissue expansion. This *in vivo* imaging and analysis technique provides capabilities not previously possible, including the measurement of *in vivo* lung function at rates of 300 fps with a spatial resolution of 330 um. This work provides greater knowledge of lung function during high frequency ventilation allowing for further research into regional lung dynamics.

## Supporting Information

Video S1 **Lung motion during ventilation at 10 Hz.** Phase contrast X-ray video showing the motion of the lung tissue as a result of the high frequency mechanical ventilation. Each frame contains 1016×1016 pixels and was recorded at 300 fps with a 2 ms exposure time.
(MP4)

Video S2 **Lung motion during 1** **Hz and 10** **Hz ventilation.** Phase contrast X-ray video showing a rabbit pup being ventilated at 1 Hz (left; 30 fps) and the same rabbit pup being ventilated at 10 Hz (right; 300 fps; video playback slowed down to 30 fps for comparison purposes). It can be seen that while there is less lung motion during high frequency ventilation, considerable motion of the lung tissue still occurs. This video highlights that due to the greater number of cycles that occur during high frequency ventilation, there is the potential for increased gas exchange with smaller lung tissue excursions. In both the 1 Hz ventilation and 10 Hz ventilation the images contained 1016×1016 pixels and were recorded with 2 ms exposure times.
(FLV)
